# Pleiotropic Effects of *Grm7*/*GRM7* in Shaping Neurodevelopmental Pathways and the Neural Substrate of Complex Behaviors and Disorders

**DOI:** 10.3390/biom15030392

**Published:** 2025-03-08

**Authors:** Beatrix M. Gyetvai, Csaba Vadasz

**Affiliations:** 1 Laboratory of Neurobehavior Genetics, Nathan S. Kline Institute for Psychiatric Research, 140 Old Orangeburg Rd., Orangeburg, NY 10962, USA; bg1551@nyu.edu; 2 Department of Psychiatry, New York University Langone Medical Center, New York, NY 10016, USA; 3 Kalymma, Stony Point, New York, NY 10980, USA

**Keywords:** behavioral genetics, addiction, complex trait, *Grm7*, mGluR7

## Abstract

Natural gene variants of metabotropic glutamate receptor subtype 7 (*Grm7*), coding for mGluR7, affect individuals’ alcohol-drinking preference. Psychopharmacological investigations have suggested that mGluR7 is also involved in responses to cocaine, morphine, and nicotine exposures. We review the pleiotropic effects of *Grm7* and the principle of recombinant quantitative trait locus introgression (RQI), which led to the discovery of the first mammalian quantitative gene accounting for alcohol-drinking preference. *Grm7*/*GRM7* can play important roles in mammalian ontogenesis, brain development, and predisposition to addiction. It is also involved in other behavioral phenotypes, including emotion, stress, motivated cognition, defensive behavior, and pain-related symptoms. This review identified pleiotropy and the modulation of neurobehavioral processes by variations in the gene *Grm7*/*GRM7*. Patterns of pleiotropic genes can form oligogenic architectures whosecombined additive and interaction effects can significantly predispose individuals to the expressions of disorders. Identifying and characterizing pleiotropic genes are necessary for understanding the expressions of complex traits. This requires tasks, such as discovering and identifying novel genetic elements of the genetic architecture, which are unsuitable for AI but require classical experimental genetics.

## 1. Introduction

Our broad, behavior-level definition of addiction is “Addiction is a multi-phase behavior. In the first phase, it produces pleasure (“reward”); thus, it is reinforcing. Then, after repeated expressions, the motivation to execute the action pattern competes with other motivations and becomes dominant—the highest priority for the organism, despite serious harmful consequences”.

Several behavioral phenotypes of addiction are known as substance use disorders (SUDs) referring to alcohol use dependence, tobacco smoking, and drug abuse (the use of cocaine, amphetamines, morphine, cannabis, phencyclidine (PCP), designer drugs, etc.), while other phenotypes of addiction, such as gambling, are not induced by self-administration of chemicals. Predisposition to addiction is heritable, generally considered as a polygenic complex trait. We believe endophenotypes of addiction should be conceptualized, diagnosed, and studied by taking advantage of the tool set of quantitative genetics, which theoretical foundations were laid down primarily by Ronald Fisher in 1918 [1] and Sewal Wright in 1926 [2]. The relevance of this theory to genome-wide association studies (or GWAS), focusing on human trait variations, has recently been discussed [3].

Because addiction is a growing global health problem, decades of intensive work and substantial US federal support of large NIH-affiliated alcohol and drug addiction research centers have been directed toward establishing therapies for substance use disorders. However, these efforts have had little impact on addiction treatment outcomes. Although our understanding of the neurobiology of addiction has greatly increased, effective treatments remain unavailable. This is concerning also because currently, more than 20 US states have legalized recreational marijuana for adults, with more states expected to follow. Kevin Sabet argued that most people who become addicted to drugs like cocaine, heroin, and fentanyl initially start with alcohol and marijuana [4,5]. 

Emphasizing the importance of the transition from occasional drug use to drug dependence, population studies show that about 15–16% of cocaine users develop cocaine dependence within 10 years of the first cocaine use, the corresponding values were 8–10% for marijuana users, and 12–13% for alcohol users [6,7]. Thus, perhaps only a fraction of exposed individuals is vulnerable to addictive drugs because of the variability in genetic, epigenetic, and environmental factors. In a substance-use-disorder multivariate genome-wide association meta-analysis of a sample of over one million individuals, nineteen independent SNPs were genome-wide significant, including nine substance-specific loci for alcohol, which may provide insight into a gene set predisposing individuals to alcohol use disorder [8].

Despite significant progress in addiction genetics, the human genetic architecture is not yet well understood. Following the principle of the evolutionary conservation of genetic and physiological features, the validity of mammalian animal models of addiction is well accepted and has been highly useful [9]. The metabotropic glutamatergic receptor subtype 7 (mGlur7, coded by *Grm7*) gene was identified in a rodent species as the first mammalian gene accountable for alcohol-drinking preference [10,11], one of the presumably large number of polygenes that interact in the determination of predisposition to addiction. Herein, we review (1) the path leading to the discovery of *Grm7* as a quantitative trait gene (QTG) in alcohol-drinking preference and (2) studies that, together, suggest a generalized role for *Grm7* in addiction and the expression of pleiotropy of *Grm7* in a variety of complex phenotypes. These call attention to greater complexity in genetic control than expected and the need for novel research strategies.

### 1.1. Review of Grm7

Herein, we review the path leading to the discovery of *Grm7* as a quantitative trait gene in individuals’ alcohol-drinking preference and studies suggesting generalized roles for *Grm7* in addiction and the expression of *Grm7* pleiotropy in various complex phenotypes. These findings highlight greater complexity in genetic control than expected and the need for novel research strategies.

#### 1.1.1. Metabotropic Glutamatergic Receptors

The chemically diverse substances of abuse share a largely common basic mechanism and neural substrate (the “reward circuit”). The most investigated nodes of the reward network are the ventral tegmental area, nucleus accumbens, hippocampus, amygdala, and prefrontal cortex. Glutamate, the predominant neurotransmitter in the brain, has two primary receptor families expressed in the reward circuitry: ionotropic glutamate receptors (iGluRs) and metabotropic glutamate receptors (mGluRs) [12].

Metabotropic glutamatergic receptors are classified into three groups based on functional characteristics and the DNA sequence of their common heptahelical domain: Group I (mGluRs 1 and 5: postsynaptic and coupled to Gq proteins), Group II (mGluRs 2 and 3: mostly presynaptic and signaling via Gi proteins), and Group III (mGluRs 4, 6, 7, and 8: mostly presynaptic and signaling via Gi proteins). Each reward circuit node expresses all the Group I, II, and III mGluRs. Group III mGluRs are receptors for the main neurotransmitters glutamate and gamma-aminobutyric acid, for Ca2+, for sweet-tasting and amino acid compounds, for some pheromone molecules, and for odorants in fish.

Two splice variants of *GRM7*, namely hmGluR7a and hmGluR7b, have been identified in the human brain. The hmGluR7a isoform, which corresponds to the human homolog of rat mGluR7, consists of 915 amino acids, whereas hmGluR7b is seven amino acids longer. mGluR7b transcripts have also been detected in wild-type mouse brains. The mGluR7 receptor is the most conserved subtype within the metabotropic glutamate receptor family, with a 99.4% sequence similarity between the human and rat homologs. Additionally, the developmentally regulated expression of mGluR7 mRNA in the human brain suggests that it plays a critical role in brain function and development in vertebrates [13].

While tunicates are not vertebrates, they are considered one of the closest invertebrate relatives to vertebrates. This is due to their possession of a notochord (at least in their larval stage), a dorsal nerve cord, and other key features that are characteristic of chordates. Evolutionary studies on ascidians (the closest invertebrate relatives to vertebrates) support the hypothesis that *Grm7* plays an important role in mammalian ontogenesis. Expression patterns of *Grm7a* and *Grm7b* at tail regression and metamorphic juvenile stages suggest their potential involvement in regulating metamorphosis in swimming larvae of the ascidian *S. clava*, a solitary tunicate species. Additionally, *Grm7a* and *Grm7b* might function as peripheral neurotransmission modulators after metamorphosis [14].

**Figure 1:**
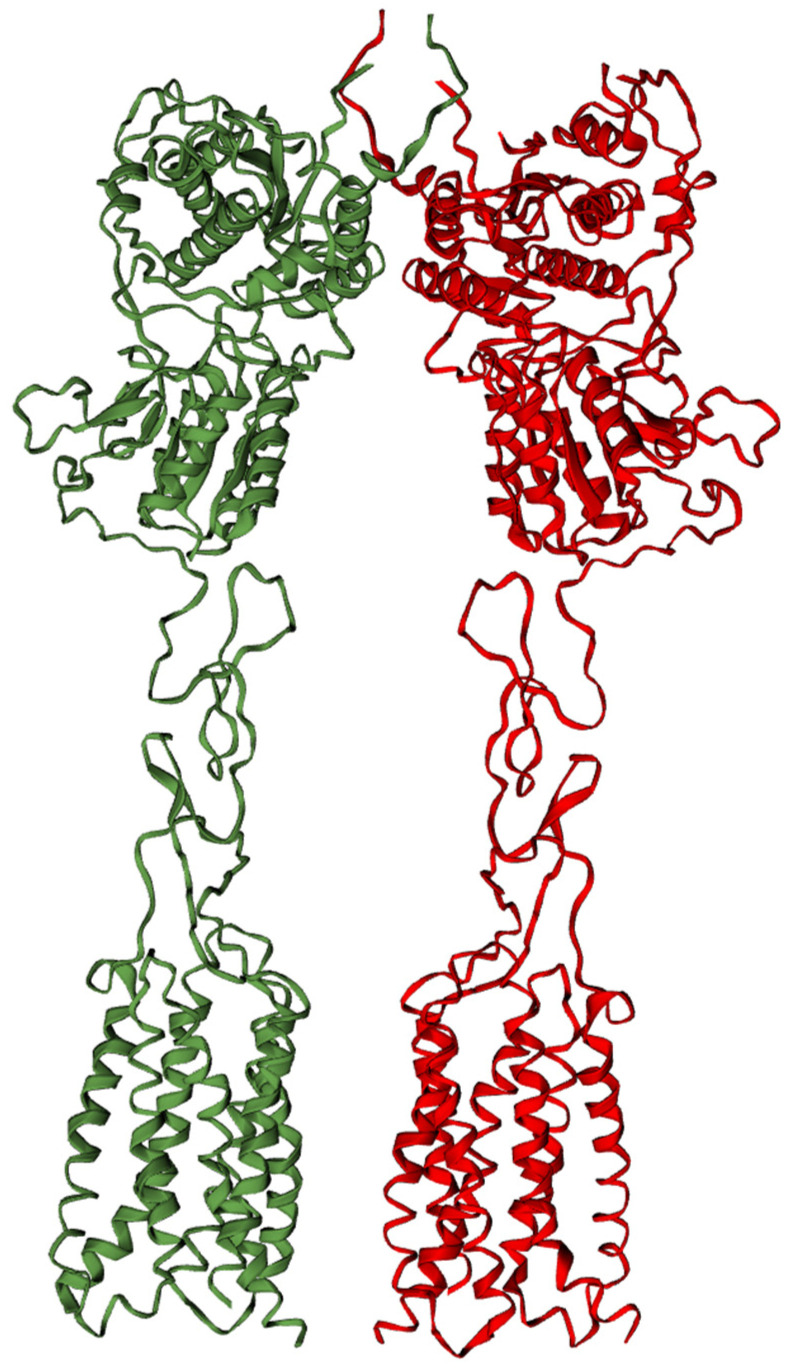
Cryo-EM structure of an inactive mGluR7 homodimer (PDB: 7EPC). The structure is colored according to the chain (chain A: red, chain B: green). From top to bottom: the Venus flytrap domain, the cysteine-rich domain, and the seven-transmembrane domain (7TM) responsible for G protein coupling. Structure is visualized using EzMol (http://www.sbg.bio.ic.ac.uk/~ezmol/ (accessed on 25 March 2024)).

Like other GPCRs, Group III receptors possess a transmembrane heptahelical domain responsible for G-protein activation. However, most Group III receptors also possess a large extracellular domain that is responsible for ligand recognition and is called a Venus flytrap module [15] (Figure 1).

The modulation of mGlu receptors’ function is complex. Homodimerization is essential for their function. These receptors are intricate allosteric proteins composed of two interacting subunits. Increasing evidence suggests that subunits of different mGlu receptors can associate to form various heterodimers. For example, in the mGlu2–mGlu7 heterodimer, the mGlu7 subunit appears to control dimeric association and G-protein activation [16]. This raises questions about tracing the mechanism of the effects of a mutation in one of the subunits on the receptor function of the heterodimer.

Group III G-protein-coupled receptors (GPCRs) operate as obligate dimers with extracellular domains that recognize small ligands, leading to G-protein activation on the transmembrane (TM) domains of these receptors by a not-well-known mechanism in which asymmetry is central to excitatory glutamate receptor activation [17].

**Figure 2:**
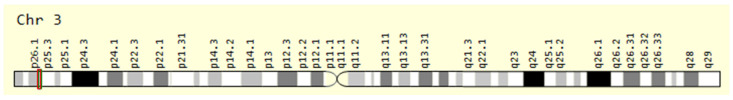
Genomic view for human *GRM7* gene on UCSC golden path. Cytogenetic band: 3p26.1 (https://useast.ensembl.org/Homo_sapiens/Gene/Summary?g=ENSG00000196277;r=3:6770001-7741533 (accessed on 29 July 2024)).

**Figure 3:**
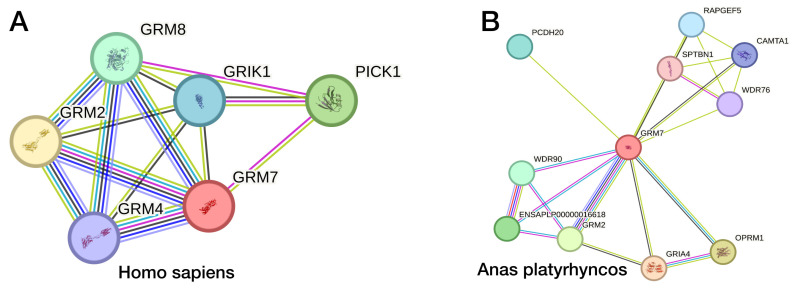
Protein–protein interaction networks for *GRM7*. Nodes represent proteins, and edges represent interactions. (**A**) Homo sapiens: Predicted functional partners of *GRM7* include *GRM2*, PICK1, *GRM8*, GRIK1, and *GRM4*. The observed interaction enrichment suggests these proteins are biologically connected, potentially indicating a role in addiction mechanisms. (**B**) Anas platyrhynchos: The *GRM7*-*GRM2* interaction is conserved in wild ducks, suggesting evolutionary conservation of this functional relationship.

The human *GRM7* gene coding for mGluR7 is located on chromosome 3, cytogenetic band: 3p26.1 (Figure 2). Human mGluR7 interacts with several proteins, including those coded by *GRM2*, PICK1, *GRM8*, GRIK1, and *GRM4*. This network has significantly more interactions than expected for a random set of proteins of the same size and degree of distribution drawn from the genome, indicating that the proteins are at least partially biologically connected. Such biological connections point to a promising research area of the mechanism in addiction, which is supported by evidence of the evolutionary conservation of biological function in vertebrates. In birds (e.g., wild ducks) the *GRM7* network shows interaction enrichment, and the *GRM7*-*GRM2* connection (as observed in mammals) is preserved (Figure 3). DNA sequence similarity suggests shared ancestry because of a speciation event; therefore, human (*GRM7* 3p26.1; Figure 2), mouse (*Grm7* Chr. 6 51.19 cM), rat (Chr. 4 q41), western clawed frog (Xenopus tropicalis) (*Grm7* chr. 4), and the unsegmented nematode (Caenorhabditis elegans) (mgl-3 chromosome 4) genes are considered as orthologs.

#### 1.1.2. Dopamine System

Genetic selection studies, based on strain differences in mesencephalic DA neuron numbers, have led to the identification of *Grm7*, a metabotropic glutamatergic receptor gene, as a factor influencing alcohol-drinking preferences in mammals.

Addictive drugs activate the mesotelencephalic dopamine system, specifically, DA neurons originating in the ventral tegmental area. These neurons project to the nucleus accumbens, where all the addictive drugs increase the DA concentration. However, their initial mechanisms of action vary. Nicotine directly activates DA neurons via nicotinic acetylcholine receptors, while cocaine elevates DA levels by blocking reuptake. Morphine and cannabis, on the other hand, bind to receptors on inhibitory GABAergic neurons in the VTA, ultimately reducing GABA release and disinhibiting DA neurons [18].

According to the discovery of genetic differences in mesencephalic dopamine neuron numbers, we initiated a genetic selection study. Replicate mouse lines were selected for high and low DA neuron numbers, using mesencephalic tyrosine hydroxylase activity (TH/MES) as an index trait. This approach was feasible for large-scale data collection because TH is the rate-limiting enzyme in DA synthesis and mouse strain differences in DA neuron numbers are attributable to the TH quantity in the mesencephalon [19,20,21,22]. Given the roles of DA in reward and addiction, inbred recombinant QTL introgression strains derived from this selection study were used to analyze voluntary alcohol-drinking preferences [11,23]. At the time these experiments began, the anatomical and functional details of the mesolimbic DA system, particularly the functional differences between the posterior and anterior ventral tegmental areas (pVTA and aVTA) and their accumbal projections, were not well understood. Later research demonstrated that cocaine and amphetamines are more rewarding when administered into the ventromedial striatum than the ventrolateral striatum, suggesting that dopaminergic neurons in the posteromedial VTA selectively project to the ventromedial striatum (medial olfactory tubercle and medial nucleus accumbens shell), while the anteromedial VTA has few projections to the ventral striatum, and the lateral VTA projects largely to the ventrolateral striatum (accumbens core, lateral shell, and lateral tubercle) [24,25].

Environmental factors can also influence the number of spontaneously active DA neurons [26]. In genetically selected alcohol-preferring, nondependent P rats, voluntary ethanol intake significantly increased the number of spontaneously active pVTA DA neurons compared to those in water-consuming controls. This indicates adaptive changes in the mesolimbic DA system of nondependent P rats, increasing pVTA DA neuron excitability and enhancing DA release in the nucleus accumbens. Regarding dependence development, it has been hypothesized that mesolimbic DA system activity increases with moderate ethanol exposure but decreases with higher, dependence-inducing levels. Moderate ethanol intake may produce adaptive changes in the pVTA that maintain ethanol consumption by increasing spontaneously firing pVTA DA neurons and raising extracellular DA levels in the nucleus accumbens in the absence of physical dependence. However, prolonged consumption and excessive intake may lead to further compensatory changes in the pVTA, such as decreased numbers of spontaneously firing VTA DA neurons and reduced nucleus accumbens DA levels during withdrawal. Drinking during withdrawal restores accumbal DA levels, at which point the organism is considered as ethanol dependent [26].

Neurobiological research on alcohol use disorder has traditionally focused on dopaminergic mechanisms. Glutamate, a non-essential amino acid and precursor for numerous nucleic acids and proteins, plays crucial roles in the central nervous system as the most abundant excitatory neurotransmitter. It contributes to development, neuronal plasticity, and memory. More recently, glutamate’s involvement in reinforcement, sensitization, learning, craving, and relapse in Alcohol Use Disorder (AUD) has been recognized, reflecting both interactions with the DA system and independent contributions, such as in reinforcement learning and context conditioning by previously neutral stimuli. The activation of glutamatergic synaptic transmission can generate memories linking substance-evoked pleasure with environmental perceptions [27].

The utility of these neural mechanisms suggests their evolutionary importance for survival, enabling organisms to locate food, shelter, and mates. Substance use disorders could thus be considered as a usurpation of this ancient reward mechanism, which is essential for life. Given that brain function relies on distributed activity, the reward mechanism is likely multi-componential. Therefore, it is unsurprising that a gene influencing substance addiction might also affect other complex behaviors (e.g., fear [28,29] and maternal behavior [30]) and behavioral disorders (e.g., epilepsy [31]). This phenomenon is known as pleiotropy. A gene affecting multiple traits is termed as pleiotropic (from Greek πλείων *pleion*, ‘more’, and τρόπος *tropos*, ‘way’).

The familial expression of addiction, with its hereditary component and complex interplay of environmental factors, like drug availability, is a subject of intensive research and diverse opinions. The extent of genetic control remains a topic of debate. Some studies, including family, adoption, and twin studies, suggest from moderate to high heritability of addiction [32]. Others highlight that most individuals can use even highly addictive drugs recreationally without developing addiction [18]. Further research is needed to explain the heterogeneity in reward system self-stimulation observed even in genetically identical mouse populations [33]. Such phenotypic variability in a homogeneous population likely arises from uncontrolled variations in developmental, maintenance, or experimental conditions. Despite these complexities, the genetic influence on addiction is generally accepted.

Recent large-scale GWAs have revealed genetic correlation between psychiatric disorders. Confirmation of the results may resolve problems in psychiatric nosology and explain the observed overlap in disorders. Genetic correlation between psychiatric disorders reflects the contribution of the whole genome. It does not reveal the link between a particular gene and a phenotype, and the lack of significant genetic correlation does not necessarily mean the lack of the effect of a single gene on phenotypes [34].

## 2. Methods

### 2.1. Search Strategy

A computerized literature search for studies investigating different paths of neurobehavioral genetic research leading to the identification of *Grm7* was performed. The literature search also included biochemical, cellular, and neurobiological studies demonstrating the function of mGluR7 or *Grm7*/*GRM7*, as well as reports on the use of artificial intelligence in targeted aspects of complex trait genetics.

### 2.2. Search Resources

Searches were performed using the terms QTL, complex trait, gene mapping, addiction, mGlur7, *Grm7*, *GRM7*, behavioral genetics, neurogenetics, AI, and GWAS in the PubMed and SCOPUS databases and Google Scholar, including “similar articles” identified by search engines, and articles cited by search-identified reports between 1 September 2023 and 1 June 2024, without limits on the language or publication date.

### 2.3. On Components of the Review: Grm7/GRM7 Contribution to “Neurological Condition” and “Neurological Disease”

The review includes sections referring to “neurological condition” and “neurological disease”. While there are general guidelines to differentiate them, the lines can blur, and the terms are sometimes used interchangeably in both medical and everyday language. The factors contributing to this vagueness include the following: An overlap of definitions, context-dependent usage, a lack of universal standards, the evolution of terminology, and patient-centered language. The distinction between a neurological disorder and a neurological condition is not always clear-cut and can depend on factors like severity, duration, underlying pathology, and the context in which the terms are used. While “disorder” often implies a more defined medical diagnosis with specific symptoms, “condition” is a broader term that can include both pathological and non-pathological states. For this review, we define “condition” as a broader term; it describes a state without necessarily implying a disease and is often used to describe a broader or temporary state of the nervous system that may not involve a pathology (e.g., a pinched nerve, transient ischemic attack, or stress-induced headaches). The term “disorder” is more specific and implies a pathological state that causes symptoms or dysfunction, for example, epilepsy, Parkinson’s disease, Alzheimer’s disease and multiple sclerosis; disorders often have a known cause or set of causes. However, we recognize that, in practice, the terms may overlap, and their usage can vary among healthcare professionals.

## 3. Neurodevelopment

### 3.1. Brain Development, Neuroprotection and Apoptosis

mGluR7 may play a positive role in glial survival involving the activation of PI3K/Akt and MAPK/ERK1/2 pathways. The protective effects of the mGluR7 allosteric agonist AMN082 in glial, neuronal, and neuronal–glial cell cultures against harmful stimuli were tested using staurosporine and doxorubicin. AMN082 alleviated glial cell damage and decreased DNA fragmentation (while the effect was mGluR7 dependent), and caspase-3 was not inhibited. Comparing glial and cerebellar granular cells derived from *Grm7*+/+ and *Grm7*-/- mice, they demonstrated a higher cell-damaging effect in *Grm7*-/--mouse-derived glia but not in cerebellar granular cells [35].

mGluR7 in Vestibular Compensation: mGluR7 has been implicated in facilitating behavioral recovery, known as “vestibular compensation”, following unilateral labyrinthectomy (UL). This process involves specific brain regions, including the medial vestibular nucleus (MVN) and the cerebellar flocculus. Zhou et al. investigated the expression patterns of mGluR2 and mGluR7 in the bilateral MVN and flocculus of rats at various stages post-UL. Their findings revealed that, in the MVN, both the mRNA and protein levels of mGluR7 were significantly reduced on the ipsilateral side on the first day following UL. This early downregulation suggests that mGluR7 may play a critical role in the initial rebalancing of spontaneous resting activity within the MVN, contributing to the early stages of vestibular compensation [36].

At present, it is not clear if mGluR7’s role in “vestibular compensation” can shed light on cellular processes and the role of heritable dysfunctional mGluR7 in age-related hearing loss. Friedman et al. performed histochemical studies on human and mouse cells and showed that mGluR7 is expressed in hair cells and in spiral ganglion cells of the inner ear, indicating that common alleles of *GRM7* contribute to an individual’s risk of developing age-related hearing loss, possibly through a mechanism of altered susceptibility to glutamate excitotoxicity [37].

Sevoflurane, one of the most used pediatric anesthetics, was found to cause developmental neurotoxicity [38]. mGluR7 has been shown to be involved, together with extracellular-signal-regulated kinase 1 and 2 (ERK1/2), in the development of sevoflurane neurotoxicity. Beta-arrestins (β-arrs) are known as scaffolds, adapters that mediate distinct intracellular signal transduction initiated by GPCR activation, and as negative regulators of GPCRs. Wang et al. showed that β-arr1 small interfering RNA (siRNA) or β-arr2 siRNA transfection can reduce the neuroprotective roles of group III mGluRs’ orthosteric agonist LAP4 and mGluR7’s allosteric agonist AMN082. Both agonists activated mGluR7, leading to significantly attenuated sevoflurane-induced neuronal apoptosis. The neuroprotective role of AMN082 is completely reversed by the ERK1/2 inhibitor 1,4-diamino-2,3-dicyano-1,4-bis[2-aminophenylthio]butadiene (U0126). In behavioral studies, LAP4 or AMN082 significantly improves the emotional and spatial learning and memory disorders induced by postnatal sevoflurane exposure [39]. Because glutamate is the major excitatory neurotransmitter in the brain, it may be expected that mGluR7 plays a role in the development of the excitation–inhibition balance of the brain. Kasatkina et al. assumed that the increased level of plasma membrane cholesterol observed under vitamin D deficiency (VDD) may influence the function of neurotransmitter receptors implicated in the modulation of the neurotransmitter release. They investigated mGluR7 by comparing the effect of MMPIP, an allosteric inhibitor of mGluR7, on stimulated [3H] GABA release from control and VDD synaptosomes. The negative allosteric modulation of mGluR7 significantly enhanced the exocytotic GABA release, which was decreased under VDD, thereby suggesting a neuroprotective effect [40].

### 3.2. Human GRM7 Gene Mutations and Consequences for Neurodevelopment and Behavioral Disorders

Supporting a role of *GRM7* in human neurodevelopmental disorders (NDD-s), studies on genetically targeted and *Grm7*-variant mice have reported symptoms similar to clinical symptoms (see Section 4.2. [31,41,42,43]).

Clinical studies have shown that in Rett syndrome (RTT, caused by a mutation in MECP2), mGlu7 receptor protein levels are diminished in the brains of patients and in an animal model of RTT [44]. Early studies on ADHD pointed to *GRM7* [45], and later, in ADHD and epilepsy, the interaction of mGlu7-Elfn1 has been implicated (Elfn1 is clustered in the carboxy-terminal region required for mGluR7 recruitment). Although Elfn1 KO mice show hyperactivity and sensory-triggered epileptic seizures, damaging missense mutations of ELFN1 were found in clinical epilepsy and attention deficit hyperactivity disorder (ADHD) studies, suggesting evolutionary conservation across species [46].

Predisposition to addiction has not been considered as a typical NDD; however, the involvement of *Grm7* in alcohol-drinking preference and the suggestive preclinical genetic associations of alcohol preference with locomotor activity and mesencephalic DA system traits may justify a closer look. The phenotypic comparison of highly inbred (isogenic) mouse strains, for example C57BL/6J and Balb/cJ, and results of RQI studies raise the possibility that the associations of higher open-field locomotor activity (hyperactivity-like behavior), higher alcohol-drinking preference (an accepted model of an aspect of SUD), lower mesencephalic-TH activity (a proxy for the DA neuron number) in the midbrain, and lower brain *Grm7* mRNA abundance in C57BL/6J are the result of complex interactions of partially overlapping gene sets [11,23,47,48,49,50,51,52]; however, rigorous experiments are required to exclude chance associations. A similar pattern could be observed in a comparison of RQI-derived congenic and *Grm7* KO mice in terms of alcohol drinking and *Grm7* mRNA expression in the brain [52].

Observations that (1) SUD and other neuropsychiatric disorders have been associated with astroglia dysregulation [53] and (2) mGluR7 may play a positive role in glial survival involving the activation of PI3K/Akt and MAPK/ERK1/2 pathways [35] may represent another bridge between preclinical and clinical work. Genetic studies linking *GRM7* to other NDDs (such as ASD, MS, and specific *GRM7* mutations associated with brain atrophy and hypomyelination, intellectual disability, developmental delay, autism spectrum disorder, and seizures) have recently been reviewed in detail [54].

#### Developmental Delay/Intellectual Disability (DD/ID), Seizure

A recent whole-exome sequencing (WES) study on consanguineous Arab families exhibiting DD/ID phenotypes identified *GRM7* with a homozygous missense mutation (c.461T>C:p.I154T) and heterozygous missense mutations (c.1972C>T:p.R658W and c.2024C>A:p.T675K) from affected siblings with DD/ID, seizures, hypotonia, and brain atrophy [55]. Further research has revealed that pathogenic mutations in *GRM7*, associated with neurodevelopmental disorders, impair axon outgrowth and the development of presynaptic terminals. Notably, defects caused by the mGlu7 I154T mutation can be reversed with agonists, offering a strong rationale for targeting mGlu7 as a potential therapeutic strategy. Additionally, this mutation has been linked to severe developmental delays and epilepsy. The mGlu7-I154T substitution results in a marked reduction of mGlu7 protein expression in both HEK293A cells and mice. Mutant mice exhibited symptoms similar to those in *Grm7* knockout mice, including impaired motor coordination, deficits in contextual fear learning, and seizures. These findings provide functional evidence that a disease-associated mutation in the mGlu7 receptor is sufficient to cause neurological dysfunction in mice, further validating the role of *GRM7* mutations in neuropsychiatric diseases. [56]. Future studies on organisms carrying the mutation can shed light on other phenotypes which have been shown to be affected in *Grm7* KO mice, such as alcohol drinking [52].

## 4. Neurological Conditions

### 4.1. Genetic Preclinical Models

#### 4.1.1. *Grm7* Pleiotropy in Preclinical Alcohol Drinking

##### The Recombinant QTL (Quantitative Trait Locus) Introgression (RQI) Approach and Identification of *Grm7* as a QTG (Quantitative Trait Gene) in Alcohol-Drinking Preferences

While there is no animal model that can account for all aspects of a human psychiatric disorder, animal models are highly useful in studying specific features of human addiction. For example, reinforcement is a significant factor in the initial phase of alcoholism. The mouse model of alcohol-drinking preference, which measures alcohol consumption in a situation where an experimentally naïve mouse can choose between water and alcohol solution, is an accepted model of the reinforcing effects of alcohol. To explore the genetic control of the mesotelencephalic dopamine system, we constructed quasi-congenic strains by Recombinant QTL Introgression RQI, which led to the first discovery of a gene (*Grm7*) accounting for alcohol preference in a mammalian model of voluntary alcohol drinking [10,11,23].

##### *Grm2* in Alcohol-Drinking Preference and Interaction with *Grm7*

Further efforts of NIAA and alcohol research centers led to the identification of the second gene (*Grm2*) involved in alcohol-drinking preference [57], and work on metabotropic glutamate receptor subtype 2 (mGluR2) expression expanded our understanding of glutamate’s involvement in addiction [58]. mGluR2, like the seven other mGluR subtypes, belongs to the class C family of G protein-coupled receptors (GPCRs) which comprise three groups (Groups I, II, and III) of mGluRs. As described above, mGluR2 is a Group II presynaptic receptor, and mGluR7 is a group III presynaptic receptor; both receptors signal via Gi proteins [59].

mGluR2 is expressed in the pyramidal neurons of the infralimbic cortex, which are particularly susceptible to the long-term effects of chronic intermittent ethanol intoxication and show a marked deficit in mGluR2 expression. Alcohol-dependent rats fail to respond to mGluR2/3 agonist treatment, as it does not reduce extracellular glutamate levels in the nucleus accumbens. These findings suggest a loss of presynaptic inhibitory feedback control. Furthermore, alcohol-dependent rats exhibit an increase in ethanol-seeking behavior, which can be reversed by restoring mGluR2 expression in the infralimbic cortex through viral-mediated gene transfer. In human alcoholics, the anterior cingulate cortex shows a significant reduction in mGluR2 transcript levels compared to control subjects, with a 2.6-fold decrease in *GRM2* transcript levels. These results suggest that the loss of mGluR2 in both rodent and human cortico-accumbal neurocircuits may be a critical consequence of alcohol dependence and a key pathophysiological mechanism driving an increased propensity for relapse [58].

As a rare example in the substance use disorder field, genetic animal model studies linked the same gene (Grm2) to alcohol consumption and preference in both species. As noted above, the report on the escalation in alcohol consumption because of the loss of mGluR2 was born from the labor of several NIAAA laboratories and large research centers. The result could be suspected by the joint interpretation of two earlier studies (1) pointing out for the first time the *Grm7*-based genetic control of alcohol consumption and preference [11] and (2) the role of mGluR7 in relapse to drug seeking, suggesting that mGluR7 activation inhibits the reinstatement of drug-seeking behavior by a glutamate-mGluR2/3 mechanism [60]. It is generally accepted that metabotropic and ionotropic glutamate receptors are potential targets for the treatment of alcohol use disorder [61].

Studies exploring the relationship between mGluR7 and mGluR2 are warranted. Following the identification of *Grm7* (mGluR7) as a modulator of alcohol consumption, Li et al. [60], using a rat relapse model, found that the systemic administration of AMN082, a selective allosteric agonist of mGluR7, dose-dependently inhibited the cocaine-induced reinstatement of drug-seeking behavior. AMN082 also suppressed cocaine-primed reinstatement, an effect that was reversed by local co-administration of MMPIP, a selective mGluR7 antagonist. Pre-treatment with AMN082 dose-dependently reduced cocaine-induced increases in extracellular glutamate levels in the nucleus accumbens (NAc) and inhibited cocaine-induced reinstatement, an effect that was blocked by either MMPIP or LY341497, a selective mGluR2/3 antagonist. These findings suggest that activation of mGluR7 by AMN082 inhibits the cocaine-induced reinstatement of drug-seeking behavior through a glutamate-mediated mechanism involving mGluR2/3 in the NAc. These findings support the idea of a pleiotropic role for *Grm7*/mGluR7 not only in alcohol drinking but also in addiction at large, and possibly in other disorders where *GRM7* is involved. Experiments on highly inbred C57BL/6By and RQI-derived congenic mice carrying natural variants of *Grm7* in a C57BL/6By background suggest genetic modulation of cocaine-induced locomotor sensitization [48] pointing to pleiotropy, i.e., the potential genetic control of addiction to other substances such as morphine and other chemicals in addition to cocaine and ethyl alcohol, by the *Grm7* polymorphism.

###### The *Grm7* Pleiotropy Hypothesis

Recently, significant genetic association between low NAc 5-HIAA and high alcohol-drinking preference in RQI strains has raised questions of the identities of underlying genes and neural mechanisms. The *Grm7* pleiotropy hypothesis proposes that genetic variation in cis-regulated *Grm7* mRNA abundance can influence mGluR7 expression and function within brain-stress circuitries. Variants of the *Grm7* gene may result in quantitative differences in the mGluR7 receptor field density. The activation of these variant fields could lead to varying levels of disinhibition in CRF-containing neurons of the hypothalamic paraventricular nucleus (PVN), which, in turn, target regions such as the dorsal raphe nuclei (DRN), among others. For example, in contrast to the alcohol-preferring quasi-congenic I5B25A RQI strain carrying the C57BL/6-type *Grm7* gene variant with lower levels of in vivo NAc 5-HIAA, alcohol-avoiding quasi-congenic C5A3 RQI mice carrying the BALB/cJ-type *Grm7* gene variant would exhibit the greater disinhibition of PVN CRF neurons and elevated CRF concentrations in the DRN. At these higher CRF levels, the normal inhibitory effects are disrupted, resulting in the uninhibited release of 5-HT in the nucleus accumbens (NAc) and constitutively elevated in vivo levels of 5-HIAA [62].

##### Roles of the mGlu7 Receptor in Responses to Psychostimulants and Opioids

mGluR7 autoreceptors or heteroreceptors function primarily at presynaptic sites to reduce the neurotransmitter release probability. Its affinity for glutamate is low: Receptor activation presumably occurs only at high glutamate concentration. Psychopharmacological investigations have suggested mGluR7 plays significant roles in responses to cocaine [60], opioid [63,64,65,66], and nicotine exposures [67] in addition to its role in alcohol drinking. The roles of metabotropic glutamate receptors (Groups I-III) in neuroplasticity following psychostimulant use disorder have recently been reviewed [68].

Studies to uncover the role of mGluR7 in addiction have included neurobiological, genetic, and psychopharmacological approaches. The latter gained impetus with the discovery of the mGlu7-receptor-specific allosteric agonist N,N′-dibenzyhydryl-ethane-1,2-diamine dihydrochloride (AMN082 [69]), which was quickly followed by a report on 6-(4-methoxyphenyl)-5-methyl-3-(4-pyridinyl)-isoxazolo[4,5-c]pyridin-4(5H)-one hydrochloride (MMPIP)—the first mGlu7-receptor-selective negative allosteric modulator [70]. Although both ligands have been tremendously useful, more information on specificity and side effects has also been collected. As a step forward in the field of mGluR7 ligands, the recent identification, optimization, and characterization of a highly potent (EC(50) 7 nM), novel allosteric mGluR7 agonist, chromane CVN636, demonstrates exquisite selectivity for mGluR7 and efficacy in an in vivo rodent model of alcohol use disorder [71] supporting the first report which links *Grm7* to alcohol drinking in a mouse model [11].

Although the above studies provide substantial evidence, the role of *Grm7*/mGluR7 in alcohol preference and consumption is often overlooked in reviews discussing the potential use of targeting the glutamate system as a novel pharmacotherapeutic approach to treating alcohol use disorders. These reviews typically focus on other key components of the glutamate system, such as the N-methyl-D-aspartate (NMDA) receptor and its specific subunits, the glycineB site on NMDA receptors (NMDAR), ionotropic L-alpha-amino-3-hydroxy-5-methyl-isoxazole-4-propionic acid (AMPA) and kainate (KAR) receptors, metabotropic glutamate receptors (mGluRs), and glutamate transporters [72].

##### Monogenic View of the Role of *Grm7* in Alcohol-Drinking Preference

The term “loss-of-function variant” is not applicable in the studied mouse strains, except for the knockout models generated by targeted mutations, because it refers to a qualitative change, a complete loss of the functional receptor protein encoded by the allele. The allele coding for the lower expression of mRNA is better described as a hypomorphic variant, where the gene product is not abolished but quantitatively diminished. Thus, *Grm7* in the inbred mouse strain C57BL/6By could be considered as a hypomorphic variant in comparison with *Grm7* in the BALB/cJ strain (cf. [61]). In general, qualitative variants lead to disrupted regulation, signal processing, or secretion, which can be detected by single-cell profiling more easily than the consequences of carrying a hypomorphic variant. Organisms with a loss-of-function variant in the heterozygous condition often can fully compensate, resulting in the lack of a phenotypic change or producing possibly subtle clinical manifestations (haplosufficiency) [73,74]. Following Sewall Wright’s reasoning, it is believed that when a quantitative allele is involved in a mechanism, the recessiveness of the variant gene indicates a compensatory mechanism, which ensures a normal phenotype. It is likely that evolution favors this kind of compensation because it enhances environmental adaptation. Also, for addiction therapy research, it can be a promising target [75]. Similarly, it is possible that a change because of a hypomorphic variation in *Grm7* has limited impact on addiction-related neural circuitries and is often compensated in individuals of randomly bred populations; however, it could be detected in special genetic preparations, such as the quasi-congenic RQI strain set with a genetically homogeneous background []. Likewise, the detection of addiction-associated *GRM7* variants may have a better chance in isolated, less diverse human populations, a consequence of the “founder effect” and the loss of genetic variation. Genetically isolated populations, or population isolates, serve as valuable resources for mapping and identifying genes, offering unique opportunities for studies aimed at better understanding the biology of common diseases and their associated traits. Well-characterized human populations provide ideal study samples for a variety of genetic investigations, including genome-wide association studies and the exploration of gene–environment interactions [76].

#### 4.1.2. Other *Grm7*-Associated Phenotypes in Preclinical Models

##### Defensive Behavior, Depression, and Anxiety

As defined by Misslin, fear can be understood as a functional defense behavior system that is part of the innate, species-specific behavioral repertoire (ethogram) essential for the survival of individuals and species. Its primary function is to protect organisms from dangerous, threatening, and aversive situations. A distinction is made between anticipatory defense behaviors triggered by potential threats and those elicited by actual dangers, particularly from predators. The neural mechanisms underlying the defense system form a hierarchical network, with the amygdala serving as the central hub for processing various threatening stimuli. The central nucleus of the amygdala projects to the midbrain periaqueductal gray (PAG), the hypothalamus, and the brainstem, which coordinate various defensive responses, including flight, defensive fighting, freezing, avoidance behaviors, submissive postures, tonic immobilization, hypoalgesia, and autonomic arousal. These circuits can be activated by either unconditioned or conditioned stimuli [77].

In mammals emotions and instincts serve as adaptive abilities for survival, most importantly, to avoid danger. Fear evokes defensive behavior, which is critical in avoiding life-threatening situations. Basic emotions (such as anger, fear, joy, sadness, disgust, and surprise) are innate, expressed in the first six months of life, affect both executive function and instinctive behavior via learning, and can activate defense behavior including the fight-or-flight response [78]. These functions are primarily regulated by the amygdala (AMY), and the evolutionary conservation of the function suggests some genetic control. mGluR7 has been detected at moderate or high intensity in the amygdala [79]. There is no generally accepted definition of “fear”. Herein, we do not consider fear as a “motivation system that activates defensive behavior” because motivation is commonly understood as a concept of intrinsic force, leading to goal-directed behaviors to serve survival and increase reproductive success—the fundamental factor in evolution. Another concept, the motivational space, has also been proposed: “the combined physiological and perceptual state, as represented in the brain is called the ‘motivational state’” [80]. Fear is an emotional response evoked by pain or stimuli associated with painful, emotionally negative events: Seeking pain and danger is usually not favored by natural selection.

Metabotropic glutamate receptor 7 (mGluR7) is expressed in brain regions implicated in emotional learning and working memory. Studies on *Grm7*-/- mice (generated at Novartis Pharma AG, Switzerland, as described by Sansig et al. [31]) have revealed deficits in taste aversion and fear responses. Although wild-type mice exhibited normal amygdala-dependent behaviors, *Grm7*-/- mice showed significantly reduced levels of immediate and delayed freezing responses to foot shocks in comparison with wild-type mice, which displayed freezing immediately after foot shocks. Further studies on conditioned taste aversion (CTA) showed an association between saccharin and the negative reinforcer LiCl, with strong CTA toward saccharin in wild-type mice. However, *Grm7*-/- knockout mice did not associate between the taste and LiCl, indicating that dysfunctional *Grm7* interferes with the expression of these amygdala-dependent phenotypes [41].

Using the Novartis knockout preparation, Sansig et al. observed a sensory-stimulus-evoked epileptic phenotype in *Grm7*-/- knockout mice which was further investigated by testing convulsant drugs, pentylenetetrazole (PTZ) and bicuculline [31]. Although *Grm7*-/+ heterozygotes were not responsive, homozygote *Grm7*-/- responded with seizures to subthreshold doses. Slice preparations suggested that mGluR7 functions as a frequency-dependent modulator. A likely role for the mGluR7a receptor and its PDZ-interacting protein (PICK1) in absence epilepsy was also investigated in rats and mice by disrupting the interaction between the receptor and PDZ proteins. This interference caused behavioral symptoms and EEG patterns that are characteristic of absence epilepsy. Deactivation of the Pick1 gene also enabled pharmacological initiation of the absence epilepsy phenotype. These outcomes indicate that disruption of the mGluR7a-PICK1 complex is enough to evoke absence-epilepsy-like seizures, thus supporting previous results [81].

Epilepsy and ADHD may also involve mGluR7 interaction with Elfn1. Missense mutations of ELFN1 were found clustered in the carboxy-terminal region required for mGluR7 recruitment [46]. Using mice, it has been shown that this interaction controls GABA interneuron synapse development. Elfn1 KO mice are deficient in recruiting mGluR7 to postsynaptic sites of somatostatin-containing interneurons in the hippocampal CA1 stratum oriens and dentate gyrus hilus, and presynaptic plasticity is also diminished.

In studies on models of complex behaviors, Novartis *Grm7*-/- and *Grm7*+/+ mice were also compared in the forced swim test, the tail suspension test (both believed to be suitable for the assessments of depression- and anxiety-like behaviors) and in four different behavioral tests, e.g., the light–dark box, the elevated plus maze, the staircase test, and the stress-induced hyperthermia test, in which the *Grm7*-/- mice showed anxiolytic-like behavior [42]—but see also [82]. These behavioral observations suggest that mGluR7 plays a role in defensive behavior in responses to aversive situations. These KO and wild-type mice were also investigated in several different learning paradigms. *Grm7*-/- mice committed more errors than WT littermates in short-term working memory tests but not in the more difficult maze tasks [83].

*Grm7*-/- mice exhibited impairments in several cognitive tasks, including scheduled appetitive conditioning, the acquisition and extinction of appetitive odor conditioning, the extinction of response suppression-based conditioned emotional responses, and the acquisition of discriminative and contextual fear conditioning. These mice were slower to establish an association between a conditioned stimulus and a positive or negative reinforcer compared to *Grm7*+/+ mice. Additionally, extinction learning of conditioned responses was slower in *Grm7*-/- mice than in wild-type animals. These delays in acquiring complex stimulus associations suggest that mGluR7 plays a crucial role in cognitive functions [84].

Human fear can be considered as an emotional reaction, resulting in a state that prepares one for defense behavior. In animal models, *Grm7* KO studies have implicated mGluR7 in defensive behaviors. Experiments by Fendt et al. focused on the formation and extinction of aversive memories. Using the mGluR7 agonist AMN082, the researchers showed that the activation of mGluR7 promotes the extinction of aversive memories in two different amygdala-dependent tasks. In contrast, reducing mGluR7 expression impaired the extinction of learned aversion. Additionally, the mGluR7 agonist AMN082 inhibited the acquisition of Pavlovian fear learning and long-term potentiation in the amygdala. The authors concluded that the role of mGluR7 in both the extinction and acquisition of fear-inducing memories suggests its involvement in anxiety [28].

Injury to the olfactory system can lead to depression. Although the exact relationship between depression and olfactory dysfunction is unclear, imaging studies show that overlapping brain areas are involved in olfactory processing and depression: the orbitofrontal cortex, the anterior and posterior cingulate cortices, the insula, the amygdala, the hippocampus, and the thalamus. There is a close anatomical link between the olfactory system and the brain circuits involved in memory and emotion [85]. With the support of clinical observations, olfactory bulbectomy (OB) has been established as an animal model of depression. OB induces changes in the levels of various protein receptors in the brain regions critical for antidepressant therapy. In line with this, Wierońska et al. investigated the effects of OB and amitriptyline (AMI) treatment on the expression of metabotropic glutamate receptors (mGluRs) in the hippocampus of mice. They found a reduction in mGluR7-IR in the OB group, which was reversed by AMI administration. However, a decrease in mGluR7 levels was observed following AMI treatment alone. These findings suggest that OB influences mGluR7 levels in the hippocampus and support the hypothesis that mGluR7 plays a significant role in the development of depression [86].

##### Pain

Pain is not classified as a psychiatric phenotype, but it is important to address in this context due to the association of chronic pain with serious mental health conditions, such as depression, anxiety, post-traumatic stress disorder (PTSD), and attention deficit/hyperactivity disorder (ADHD). Nearly half of adults with chronic pain also have one or more of these comorbid conditions. Among individuals with chronic pain, those with co-occurring mental health disorders tend to experience greater pain intensity, higher levels of disability, and a lower quality of life compared to those without such conditions. Furthermore, chronic pain can exacerbate mental health symptoms, including fatigue, sleep disturbances, difficulties concentrating, and anhedonia. Most existing research on the intersection of chronic pain and mental health conditions has focused on the relationships between chronic pain and depression, anxiety, and PTSD [87]. However, recent studies have begun to explore the links between ADHD symptoms, diagnosis, and chronic pain [88].

Depression and pain are both complex traits, and the links between them or the extent of overlap are not well known. It is generally assumed that pain can affect mood, and chronic pain can cause several problems that can lead to depression, such as trouble in sleeping and stress. The involvement of *Grm7* in the endophenotypes of defense behavior may serve as a link and target for further research.

Emerging evidence suggests that metabotropic glutamate receptors (mGluRs) play a role in sensory processing. Furthermore, the distinct distribution of the mGluR subtype mRNA in specific thalamic nuclei of normal rats indicates that these receptors may be involved in the processing of somatosensory information. In mono-arthritic rats, which display behavioral and physical signs of painful arthritis, mGluR7 mRNA expressions in the ventrobasal (VB) complex and posterior thalamic nuclei (Po) significantly decrease, as shown by in situ hybridization in selected brainstem and thalamic nuclei at different time points. The reduced expressions of mGluR7 transcripts in VB and Po may contribute to counteract the increased noxious input arising from the periphery [89]. In a rat model of inflammatory hyperalgesia and allodynia evoked by noxious input arising from the periphery, AMN082 attenuates thermal hyperalgesia and allodynia. In the paw incision model, AMN082 inhibits thermal hyperalgesia, but not allodynia. Enhancing endogenous mGluR7 activity inhibits post-injury stimulus-evoked hypersensitivity [90].

mGluR7 activation by allosteric agonist AMN082 in the spinal cord of a novel formalin model of nociceptive behavior in female sheep significantly inhibits both early- and late-phase formalin-induced hyperalgesia and pain behaviors. AMN082 also induces a rapid but short-lasting analgesia in naïve subjects. These data suggest that enhancing endogenous mGluR7 activity in the spinal cord, using AMN082, blocks pain and hyperalgesia [91].

In line with the above pain studies, in a mouse model of neuropathic pain, Osikowicz et al. demonstrated that AMN082 can attenuate allodynia (von Frey test) and hyperalgesia (cold plate test), as measured on day seven after chronic constriction injury (CCI) to the sciatic nerve, and mGluR7 is involved in injury-induced plastic changes in nociceptive pathways [92]. In contrast, in the hot plate test and tail flick test, mice treated with the mGluR7 agonist AMN082 did not show an analgesic effect but induced hyperalgesia in response to thermal nociceptive stimuli [93].

AMN082 reduces spinal withdrawal reflex thresholds and increases both audible and ultrasonic vocalizations triggered by a brief compression of the knee in the mono-arthritic pain model. It also decreases open-arm preference in the elevated plus maze (EPM) test, suggesting anxiety-like behavior. However, in arthritic animals, AMN082 does not modulate these increased spinal reflexes, vocalizations, or anxiety-like behavior. These results indicate that in naïve animals, mGluR7 facilitates pain responses and has anxiogenic (pain-enhancing) properties, while in arthritic pain models, mGluR7 fails to inhibit nocifensive behaviors or anxiety.

Further research has explored the differential effects of mGluR7 and mGluR8 activation on pain-related synaptic activity in the amygdala. In a rat arthritis pain model, patch-clamp recordings from brain slices measuring monosynaptic excitatory postsynaptic currents (EPSCs), mono- and polysynaptic inhibitory synaptic currents (IPSCs), and synaptically evoked action potentials (E-S coupling) in the laterocapsular division of the central nucleus of the amygdala (CeLC) revealed that AMN082 increases EPSCs and E-S coupling in slices from normal rats but not in pain models. This suggests that mGluR7’s facilitatory effects are indirect and depend on action potential-driven network activity. AMN082 also decreases the number of IPSCs evoked and the frequency, but not the amplitude, of spontaneous and miniature IPSCs in slices from normal rats. Thus, mGluR7 inhibits inhibitory synaptic transmission to regulate glutamatergic transmission to CeLC neurons under normal conditions, but not during pain.

In the spared sciatic nerve injury (SNI) mouse model of pain, MMPIP, a negative allosteric modulator of mGluR7 increases thermal and mechanical thresholds and promotes open-arm choice in the EPM. MMPIP also reduces immobility in the tail suspension test, as well as the number of marbles buried and digging events in the marble burying test. Additionally, MMPIP improves cognitive performance and restores the balance between the excitatory and inhibitory responses of pyramidal neurons in the prelimbic cortex of SNI mice.

Altogether, these findings show that mGluR7 NAMs reduce pain responses and affective/cognitive impairments in neuropathic pain conditions [94]. Recent reviews have summarized studies on group III mGluRs in animal models of chronic pain [95,96].

#### 4.1.3. The Role of *Grm7* in Cocaine, Opioid, Nicotine, and Methamphetamine Abuse

After pointing out the involvement of mGlurs in cocaine addiction [97,98] and the identification of *Grm7* as a gene modulating alcohol drinking [11], relapse to drug seeking was also investigated [60]. Pretreatment with AMN082 blocked cocaine-induced reinstatement, which was blocked by a selective mGluR2/3 antagonist, suggesting that mGluR7 activation inhibits the cocaine-induced reinstatement of drug-seeking behavior by a glutamate–mGluR2/3 mechanism in the NAc. Genetic studies have indicated that cocaine-induced sensitization is linked to the distal chromosome 6 region in a congenic mouse model, suggested a role for the mGlu7 receptor in the control of neurobiological responses to cocaine, and put forward the hypotheses that (1) natural variants of the *Grm7* gene show pleiotropy and can modulate cocaine-induced behaviors in addition to alcohol consumption and that (2) interactions between mGluR7 expression and estrogen receptors and estradiol may explain phenotypic variation in females. The heritable variation in *Grm7* affects cocaine-induced sensitization in female mice [48]; thus, it may affect vulnerability to substance abuse in women. These findings support the potential use of mGluR7 agonists for the treatment of cocaine addiction [60] and AUD.

### 4.2. GRM7 in Human Neurological Conditions

#### 4.2.1. Alcohol Use Disorder (AUD)

The polygenic AUD phenotype can be affected by many genes, each contributing to the phenotype with a distinct effect size and via a different mechanism. These factors can operate at the levels of genetic and epigenetic changes, mRNA abundance and sequence, protein quantity, function, structural interaction, cellular function, organ function, and whole organism, eventually leading to behavioral disorder. The effect of *Grm7* on alcohol -drinking preference is cis-regulated in our preclinical model; however, targeted in-depth clinical analysis confirming cis-regulation and evidence of evolutionary conservation across species remain to be reported.

In a recent human genetic study, we aimed to evaluate evidence for associations between *GRM7* and alcohol-consumption-related behaviors, using a single-nucleotide-polymorphism (SNP) approach as well as a gene-based approach. A study examining two SNPs in *GRM7* for their potential association with alcohol consumption was conducted using 1803 non-Hispanic European Americans (EAs) from the Colorado Center on Antisocial Drug Dependence (CADD) and 1049 EA subjects from an independent replication sample from the Genetics of Antisocial Drug Dependence (GADD). Two family-based association tests, FBAT and QTDT, were used in the analysis. In the CADD sample, rs3749380 showed a suggestive association with alcohol consumption, with the minor T allele conferring increased risk. However, no association was found in the GADD sample. A gene-based test, incorporating four Genome-Wide Association Studies (GWAS), found no significant link between variations in *GRM7* and alcohol consumption. Several limitations may account for the lack of significant findings: the selected SNPs likely do not tag expression quantitative trait loci (eQTLs), the human alcohol consumption phenotype used in the study complicates the interpretation when compared to rodent studies suggesting a cis-regulatory link between alcohol preference and *Grm7*, and the gene-based test only included common SNPs imputed across all four datasets. These limitations highlight that rare variants, potentially significant common signals within the gene, and regions further upstream were not explored, underscoring the challenges faced in complex trait genetics [99].

**Figure 4:**
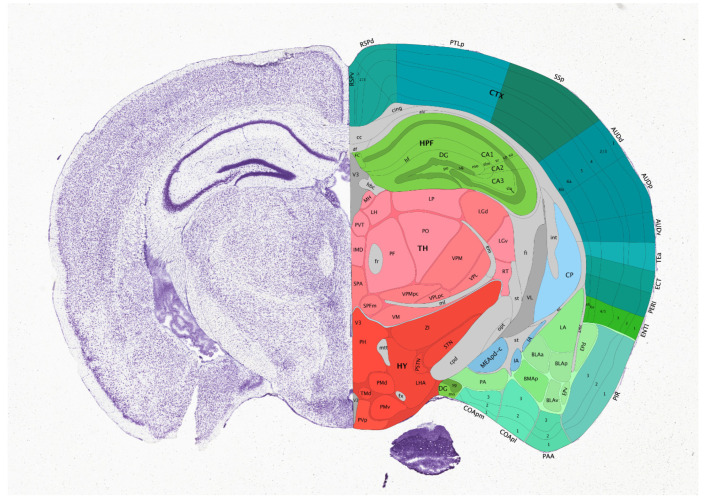
In a mouse brain CA1 metabotropic glutamate receptor 7 mRNA expression was significantly higher in congenic B6By.C6.137.54 male mice in comparison with background C57BL/6By males carrying different variants of *Grm7*. The higher mRNA abundance was associated with a significantly lower voluntary alcohol-drinking preference [52]. The position of the CA1 field (Green, HPF CA1) is shown here in the Allen Brain Atlas. Interactive Atlas Viewer approximates the position of CA1 at Bregma −2.18 [P56, coronal image 76 of 132, zoom 5.94%] (http://atlas.brain-map.org/atlas (accessed on 30 July 2024)).

Looking at gene-variant-dependent functional effects, such as those known in Mendelian medical genetics (without touching the important area of non-allelic interactions), we can gain further insight [75]. Vertebrates are diploid organisms, having maternal and paternal copies of genes. Differences between the copies can lead to allelic interactions, which manifest in molecular, physiological, behavioral, etc., phenotypic differences. In the preclinical field, the autosomal *Grm7* gene variation can serve as an example for quantitative molecular and behavioral differences: Two copies of the *Grm7* gene from the homozygous BALB/cJ mouse strain, when compared to *Grm7* derived from the inbred C57BL/6By strain, determine the higher abundance of *Grm7* mRNA in various brain regions and are associated with lower alcohol-drinking preference (Figure 4) [11,52].

Decades ago, when human genetic analysis GWAS was considered as a questionable newcomer, family-based linkage analysis dominated the field, and large centers were substantially supported by NIH in the hope that collaborating centers could identify gene variants and genetic mechanisms that predispose one to alcoholism. In spite of the facts that the number of genetic (microsatellite) markers was limited and the subject sample size was disheartening (especially considering current standards), some of the studies were influential at the time. Genotype and clinical data were used in family-based parametric and non-parametric linkage designs, candidate gene studies, and, eventually, in GWAS [100,101]. The goal of linkage studies was to detect the co-segregation of chromosome regions and alcohol-drinking phenotype, an approach with similarities to QTL mapping in preclinical models. The linkage approach led to the identification of regions on chromosome 4q23, which harbored the alcohol dehydrogenase genes (e.g., ADH1B), as well as a cluster of genes on 4p12, encoding gamma aminobutyric acid receptor genes linked to a quantitative EEG phenotype and AUD [102,103,104].

In general, candidate gene studies have been attractive because existing data could be used to develop hypotheses and, at the first glance, promised clear answers to sometimes biased human assumptions regarding the involvement of a favorite gene in AUD or other complex traits. However, in behavioral genetics, a substantial portion of proposed candidate genes has not been confirmed. A recent review of the early results of the Collaborative Study on the Genetics of Alcoholism (COGA) candidate gene studies provided information on the tachykinin receptor, bitter taste receptor genes, and ADH1B, GABRA2, CRHR1, CHRM2, *GRM8*, DRD2/ANKK1, HTT, OPRK1 and PDYN, ACN9 NFKB1, GABRR1, GABRR2, CRHR1, SGIP1, CHRNA5, NPY and SNCA see [105].

As to brain function, genes were identified in COGA linkage families, using neurophysiological phenotypes that were indices of the risk for AUD. There is a linkage to and an association between theta-event-related oscillations (EROs) and targets in the visual oddball task and SNPs in *GRM8* (the metabotropic glutamate receptor gene) that were also found to be associated with alcohol dependence and related phenotypes (e.g., depression) [106]. Functional genomic studies on COGA elucidate changes induced by alcohol-use-disorder-risk genes, using multimodal approaches with human cell lines and brain tissue, with the goal of the integration of multimodal data within COGA participants to reveal mechanisms linking genomic variants with alcohol use disorder and potential targets for future treatments. *GRM7*-related mechanisms have not been reported [107].

#### 4.2.2. Mood Disorders and Attention Deficit Hyperactive Disorder

*GRM7* variants have shown allelic associations with depression in several studies. A *GRM7* intronic SNP is strongly associated with major depressive disorder (MDD) in a GWAS of 3957 cases and 3428 controls (*p* = 1.11 × 10^−6^. These findings were supported in a further analysis using a narrower phenotype (N = 2191), suggesting that *GRM7* is a strong MDD candidate gene [108]. Resequencing the *GRM7* gene in 32 bipolar samples and 32 random controls selected from 553 bipolar cases and 547 control samples (UCL1) provides additional evidence. In a second sample of 593 patients and 642 controls (UCL2) the association between *GRM7* and bipolar disorder (BP) could be confirmed. Kandaswamy et al., demonstrated that rs1508724 and rs6769814 in *GRM7* are significantly associated with bipolar disorder (BP). DNA sequencing revealed mutations in three cases, which were absent in control subjects, and a 3′-UTR SNP (rs56173829) in *GRM7* was significantly associated with BP (*p* = 0.035; OR = 0.482). The presence of deletions and a duplication within *GRM7* provides further support for the pre-existing evidence that copy number variants in *GRM7* may have a role in the etiology of BP. Interestingly, bioinformatic analyses predicted a change in the centroid’s secondary structure of RNA and alterations in the miRNA binding sites for the mutated base of rs56173829 [109]. These support the evidence for selective microRNAs and their effectors as common long-term targets for the actions of mood stabilizers and that endogenous miR-34a regulates *GRM7* levels [110]. Other studies could not confirm an association between the *GRM7* polymorphism rs162209 and depression in 479 patients with depression and 329 normal controls [111].

*GRM7* variants have been associated with numerous psychiatric disorders, reflecting the prominent excitatory neurotransmitter role of glutamate in the brain. The CC genotype of rs6782011 in *GRM7* is significantly associated with BPD2 (bipolar disorder 2) in a recessive model (OR (95% CI) = 1.78 (1.09–2.91), adjusted *p*-value = 0.04) and with ADHD in dominant and co-dominant models (OR (95% CI) = 1.98 (1.11–3.53), adjusted *p*-value = 0.04; OR (95% CI) = 2.27 (1.23–4.17), adjusted *p*-value = 0.04, respectively) in an Iranian population of 364 patients with mood disorders (BPD1, BPD2, and MDD), 250 age-matched controls, 108 ADHD patients (males = 81; females = 27), and 164 age-matched controls (males = 123; females = 41) [112]. These results support earlier results by Mick et al.: An “intriguing association among suggestive findings (rs3792452; *p* = 2.6 × 10^−5^) with *GRM7*, as it is expressed in brain structures also previously associated with ADHD” [45].

#### 4.2.3. Age-Related Hearing Impairment (ARHI) (Presbycusis)

The first whole-genome association study for ARHI was performed using 846 cases and 846 controls collected in European countries. Then, a selected group of SNPs (n = 23) was individually genotyped in an independent European replication group (138 samples), identifying a highly significant and replicated SNP located in *GRM7*. More recent reports confirm the role of *GRM7* and point out that human age-related hearing loss is a complex trait. Variants of genes *GRM7*, ISG20, TRIOBP, ILDR1, and EYA4 have shown significant associations with hearing loss and highlight the need to disentangle the genetic architecture of ARHL and standardize phenotyping methods for facilitating data sharing and collaboration across research networks [113]. 

#### 4.2.4. *GRM7* Natural Variants in Human Physiology and Behavior

*Grm7* orthologs and paralogs are involved in basic organismal development. Looking at the history of the specific aspects of human neurobehavioral development, the *DSM-III* defined autistic disorder as a developmental disorder. About a decade later, the *DSM-V* grouped a set of conditions together, such as intellectual disability (ID), autism spectrum disorder (ASD), attention-deficit/hyperactivity disorder (ADHD), tic disorders, and specific learning disorders, because of comorbidity, phenotypic overlap, and support by genetic data. It has been hypothesized that ID, ASD, ADHD, schizophrenia, and bipolar disorder lie on a neurodevelopmental continuum [114]. 

## 5. Neurological Disorders

### 5.1. Association of GRM7 with Other Human Phenotypes: General Cognitive Ability, Alzheimer’s Disease

When evaluated with a five-component cognitive score, which includes tests of verbal fluency, forward and backward digit span, and immediate and delayed recall, the heritability of cognitive function ranges from 50% to 80%. This heritability varies over the course of development, increasing from childhood to around 65 years of age, and then remaining above 50%, indicating a significant genetic contribution to individual differences in cognitive performance. A GWAS of cognitive function was conducted on individuals aged 90.1 to 100.8 years, and a suggestively significant SNP was mapped to *GRM7* (rs28502528, *p* = 3.74 × 10^−7^). This phenotype significantly influences individuals’ health and socio-economic status; therefore, understanding its genetic architecture and its biological substrate is highly desirable [115]. As can be anticipated based on the cognitive ability results, *GRM7* is also associated with Alzheimer’s disease [116].

### 5.2. Hypertension

Longitudinal analyses from the Genetic Analysis Workshop 19 (GAW19) examined genome-wide association (GWA) and whole genome sequencing (WGS) data across up to four time points for blood pressure-related phenotypes. The statistical models employed included generalized estimating equations (GEEs). This approach offers a robust strategy for understanding changes in long-term averages and shifts in complex disease phenotypes over time, facilitating more accurate definitions of disease status and a deeper understanding of the trajectory of traits and disease progression. In GAW19, the GEE-based model used to identify gene-based associations with four hypertension-derived phenotypes pinpointed a significant locus, *GRM7*, which passed multiple correction tests for two hypertension-related traits [117]. Studies on neurodevelopment-based psychiatric disorders could benefit from such longitudinal analytical approaches if carried out with comparable precision and reproducibility.

### 5.3. Cancer

Genetic studies of samples from several forms of cancer recognize *GRM7* as one of the contributing genes. Studies of benign and tumor prostate samples from African American men show the differential methylation of *GRM7*, which is significantly associated with cancer progression [118]. The genes of an integrative nine-gene multi-omics signature mapped in several chromosomal regions, including 3p with copy number alterations in *GRM7*, correlated with head-and-neck-cancer (HNC) patients’ survival, suggesting that copy number alterations in *GRM7* can predict HNC patients’ survival [119].

About 50% of the genes are dysregulated in glioblastoma multiforme (GBM), which is one of the most aggressive forms of cancer. *GRM7* is one of a subset of genes that predict a positive prognosis in low-grade gliomas and a negative prognosis in GBM, showing progressive downregulation across glioma grades implicating *GRM7* in cancer progression [120].

## 6. Psychiatric Disorders

###  Metabotropic Glutamate Receptor Genes in Schizophrenia (SCZ)

SCZ is often associated with SUD, potentially reflecting common pathophysiology and risk genes [121]. SCZ is occurring at a prevalence of approximately 1%. A search of common gene variants and disruptive mutations as factors in polygenic determination and rare heterogeneous expression, respectively, has not identified a definitive connection between mGlu receptor genes and SCZ [122]. The clinical research approach evolved from candidate gene studies to GWAs and next-generation sequencing (NGS); still, the lack of genetic-evidence-based progress in SCZ treatment suggests that further significant improvement, perhaps a paradigm shift, is needed. This may include the consideration of (1) implementing a precision medicine approach, “an emerging approach for disease treatment and prevention that takes into account the individual variability in genes, the environment, and the lifestyle of each person”, i.e., population genetic aspects of predisposition: The significance and frequency of genetic factors can be different in populations of European, African, or Asian ancestries (cf. [123]). This reflects a long-standing understanding in animal genetics: Sometimes, different genetic architectures in different inbred populations may yield similar phenotypes based on different genes, interactions, and physiologies. (2) Addressing several critical issues in pleiotropy mapping for psychiatric disorders [vide [34,124]]: (a) the implementation of a pleiotropy-centric analysis, (b) the simultaneous examination of a much larger number of psychiatric disorders as a high-dimensional problem to gain comprehensive insight into shared genetic components (and, perhaps, into gene–gene interaction patterns) underlying distinct disorders, and (c) the disentanglement of horizontal or vertical pleiotropy to evaluate the potentially causal association among distinct psychiatric disorders, using Mendelian randomization (MR) methods. (3) Investigating cis-regulation in conjunction with brain single-cell genomic technologies, which provide unprecedented resolution and throughput to measure the transcriptomic and epigenomic profiles of individual cells [125].

*GRM7* was first associated with schizophrenia in a study of Japanese patients with schizophrenia in a case-control study of 2293 Japanese patients and 2382 control subjects, examining the influence of one polymorphism associated with schizophrenia on the expression of *GRM7* in a dual-luciferase assay (in transfected cells, mutations in all the exons, exon/intron junctions, and promoter regions); the results suggested a potential association of a synonymous polymorphism (371T/C, rs3749380) in exon 1 with schizophrenia [126]. Further studies on schizophrenia with polymorphisms in *GRM7* identified two neighboring SNPs (rs12491620 and rs1450099) in *GRM7*, showing a highly significant haplotype association. Additional typing of the two SNPs, using an expanded sample set (404 cases and 420 controls), confirmed the significant association with the disease. These data suggest that at least one susceptibility locus for schizophrenia is located within or nearby *GRM7* [127]. A larger study on the Asian population (1034 schizophrenic patients and 1034 healthy controls of Chinese Han origin) confirms the *GRM7* schizophrenia association. SNPs rs13353402 and rs1531939 demonstrate a significant difference between schizophrenic patients and control subjects in allele frequencies (rs13353402: *p*-value = 0.0307; rs1531939: *p*-value = 0.0328), indicating that the *GRM7* SNPs rs13353402 and rs1531939 might be associated with schizophrenia in the Chinese Han population [128].

Although according to the Schizophrenia Working Group, *GRM7* is not reflected among the “credible causal schizophrenia SNPs, coding variants, and eQTLs” [129], following and expanding upon the research on schizophrenia in the Chinese Han population, the interactions between two Group III mGluR genes, *GRM7* and *GRM8*, were studied in a population with SCZ and major depressive disorders (MDD) in samples of 1235 SCZ patients, 1045 MDD patients, and 1235 normal controls. The data show that rs2229902 of *GRM7* and rs2237781 of *GRM8* are significantly associated with SCZ. Moreover, rs779706 of *GRM7* and rs1361995 of *GRM8* are associated with MDD in the presence of between-gene interactions [130].

Copy number variation (CNV) research offers a unique perspective toward understanding disease mechanisms. It is reflected in genomic imbalances and often linked to neuropsychiatric disorders; however, there is no significant evidence supporting a role for CNV in SCZ [131,132].

For translational research, pharmacogenomics is an important area because it can guide the clinical management of schizophrenia. Although GWAS suggests that *GRM7* has potential risk variants for schizophrenia, the required significance level has increased; the relationship between the *GRM7* variants and the risk of schizophrenia is still uncertain, and there are significant individual variations in responses to antipsychotic drugs. *GRM7* variants may predict the risk of schizophrenia and antipsychotic effects of common drugs: rs1516569 in *GRM7*, a significant risk factor (OR = 0.95, *p* < 3.47 × 10^−4^), and rs9883258 in *GRM7* (OR = 0.84, *p* = 2.18 × 10^−3^) are potential biomarkers for therapeutic responses of seven commonly used antipsychotic drugs (aripiprazole, haloperidol, olanzapine, perphenazine, quetiapine, risperidone, and ziprasidone) in the Chinese Han population. Other SNPs of *GRM7* such as rs779746 (2KB_upstream_variant, upstream_transcript_variant, and intron_variant), rs480409 (intron_variant), rs78137319 (non_coding_transcript_variant, coding_sequence_variant, and synonymous_variant), and rs1154370 (intron_variant), are significantly associated with treatment responses with the above antipsychotic drugs [133]. Additional SNPs of *GRM7* (rs141134664, rs57521140, and rs73809055) are associated with risperidone treatment outcomes in a schizophrenia GWAS. These, and similar, studies demonstrate that pharmacogenomics has the potential to facilitate precision medicine in schizophrenia treatment [134].

## 7. Conclusions

### 7.1. Basic Studies

Preclinical and clinical studies suggest that behavior-level pleiotropy is manifested via complex interactions between *Grm7* and a variety of genes at the cellular level; however, the exact gene and neuronal networks and the extent of overlaps remain to be mapped out.

Historically, success in attempts to identify the genetic variants underlying addiction and other complex traits has been limited partially because of researcher bias in the selection of candidate genes and the inadequate power of linkage studies. It should serve us as a reminder that with naïve confidence, the function of noncoding DNA, which constitutes the majority of the genome (about 98% in Homo sapiens), was first casually referred to as “junk DNA” in the 1960s and then accepted generally after the 1970s based on the hypothesis that the genome necessarily contains noncoding DNA passively accumulated over millions of years [135]. This way of thinking is slowly changing with progress in DNA and RNA sequencing leading to hypothesis-free microarray studies in functional genomics and GWAS in genetics. It has become clear that noncoding DNA can regulate protein production and affect phenotypes, including behavioral disorders [e.g., [136]], and repeated sequences help to maintain the integrity of chromosomes.

### 7.2. Clinical Studies

Recently, extensive collaborations have allowed GWAS with very large sample sizes and novel advancements in high-throughput sequencing technologies in a genotyping-by-sequencing approach to open up new horizons for extensive genotyping; however, to yield valid results, they require independent confirmation and consistent, reliable phenotyping and endo-phenotyping. Furthermore, the collected data require novel, robust multi-dimensional methods of information processing and engagement with the recent genetic revolution in neuroscience, single-cell RNA sequencing (scRNA-seq) [125]. 

Finally, as the field of complex trait genetics progresses, it is expected that psychiatric nosology will change to resolve current problems in diagnosis and treatment and reflect the genetic reality of pleiotropy, interactions, and genomic-based brain function. 

## Data Availability

No data were used for the research described in this article.
